# New forms of artivism in relation to contemporary societies. Tuty Moreno Campos’s *soft artivism*

**DOI:** 10.3389/fsoc.2025.1548970

**Published:** 2025-06-17

**Authors:** Sofia Sousa

**Affiliations:** Faculdade de Letras da Universidade do Porto, Instituto de Sociologia da Universidade do Porto, Porto, Portugal

**Keywords:** soft artivism, migrant women, artivism, artistic practices, Global South

## Abstract

With this article, we intend to look at the intersection between the experiences of women artists, particularly in terms of their individual and collective practices, in relation to the creation of art products with an artivist or art-based activism slant. Based on the artistic practice of Tuty Moreno Campos - a migrant artist - we intend to highlight and discuss a new type of art-activist work, in other words, to discuss the emergence of concepts such as soft artivism. To do this, we used a qualitative methodology based on techniques such as semi-structured interviews and content and visual analysis.

## 1 Beyond traditional artivism

Reaching the breadth of women’s artistic creations in contemporary times is challenging. In fact, little is known about the intersection between the experiences of women artists, namely at the level of their individual and collective practices in relation to the creation of artistic products with an artivist or art-based activism bent during the migrant experience (Martin, 2022). With the elaboration of this article, we intend to analyze and discuss – albeit in an exploratory way – a new typology of artistic-activist works, that is, it is our intention to discuss the emergence of artistic works that materialize the concept of soft artivism^1^, more specifically the works of Tuty Moreno Campos.

In our assertion and in the context of this article – referring to the artistic productions of Tuty Moreno Campos – the concept of soft artivism, refers to a form of advocacy or activism that emphasizes non-confrontational, gentle, or subtle approaches that wish to promote other forms of social or political change. Unlike traditional or more confrontational forms of activism that may involve protests, marches, or direct action, soft activism often focuses on awareness-raising, education, community-building, and collaboration, and it’s an especially active practice in connection to the digital world.

The main objective of this article is to contribute to the theoretical-conceptual advance around the broad notion of artivism in contemporary societies (Chaia, 2007; Vieira, 2007), evidencing the emergence of different typologies of artistic-activist creation, which start from an intimate/sensitive place of performance/creation and are anchored in performances and the physical body to grant alternative spaces of expression.

Thus, this article focuses on the intersectionality of the themes of artistic creation and contemporary artivism(s), exploring the specific case of the artist Tuty Moreno Campo and two of her works: the photo-performance *Vaciarce* (2019) and the video-performance *Árido* (2020). These artistic-artivist-creative projects were selected because they present a multidisciplinary approach, but also because in both, the artist’s body is in evidence, denoting a modality of soft artivism.

It is also important to assess as a form of framing that since the 1970s, the feminist movement has used artistic practices as a form of resistance and vindication of public space, in a logic in which the personal and the political are intertwined. This issue is explicit in the adage ‘the personal is political’ that we will develop later. Thus, we want to demonstrate that the soft artivism of artists such as Campos does not break with the heritage of feminist practices, but rather configures itself as a continuity, marked by the emphasis on sensitive experience and intimate transformation.

When we think about artivism in the context of Latin America and relate it to the issue of gender, there is a tendency to focus on the theme of femicide (Fischer, 2017; Wieczorek, 2023) and colonialism (Robinson, 2015; Martinez, 2018; Sandoval and Latorre, 2008) which, although extremely relevant, does not encapsulate the reality of all the countries that make up Latin America nor the reality of all Latin American women; neither do they fully circumscribe the female artistic practices from Latin America. Also, in the 60’s, art was used by feminist groups as a means of social denunciation of the gender inequalities experienced. This idea remains but expands in terms of platforms, discourses and strategies of creation and dissemination, i.e., do-it-yourself praxis (Guerra and Sousa, 2023), social networks (Kara, 2015), Artifical Intelligence (Suárez Val et al., 2023), etc.

To provide some framing to Campos^2^ artistic we state that she is a visual artist and performer, born in Argentina, in the city of Buenos Aires, who currently resides in Mexico, in Mexico City. Throughout her career, she has received several research and artistic creation grants from public and private institutions in Argentina, Mexico City and Estonia. She has also participated in several collaborative exhibitions in countries such as Mexico, Canada, Spain, Colombia and Argentina (Maiztegui-Oñate et al., 2022).

In her work, she experiments with different disciplines. Through the interview we made to her, we inferred that she’s interested in addressing the dialog between body and disease, for which she uses video-performance as an intimate place of action. She uses it to become aware of the body, to give rise to emotional states that manifest in it, to represent them, to go through them and, if possible, to transmute them. She operates with materials and sounds taken from everyday life, which are considered precarious, discarded, violated elements, such as sounds produced by the body (moaning, pain, discomfort, etc). At the same time, in her creations she uses drawing and painting to propose ruptures of language and space and installations/performance are a way of creating environments that include fractures that form a kind of refuge. Her practice is a continuous exercise where ruptures and reconfigurations are emphasized as echoes of existence itself.

Thus, this article is structured in five sections, in addition to this introductory one. We move on to a methodological section, where we present the techniques used, namely visual sociology, content analysis and semi-structured interviews. We also have another section where we address the theme of contemporary artivist practices (soft artivism), followed by two sections which are directed to the discussion of two artistic works by Moreno Campos, i.e., *Vaciarce* and *Árido*, and their intersection with the problems of the body, soft artivism, territories and performance. We end the article with some final remarks and future research directions.

## 2 Methodological fragments

This article was built through the empirical materials that were collected through two sources, namely the performances and artistic creations of Tuty Moreno Campos, and the semi-structured interview that was conducted with her. We used a qualitative methodology that was accompanied by an exercise of visual sociology (Harper, 2005), and the technique of content analysis was applied to both collected materials (interview and artistic works). For the analysis of both the photo-performance, the video-performance and the interview, categories and subcategories of analysis were created, in line with the content analysis technique (Murthy, 2024; Cho and Lee, 2014).

This research was motivated by the need to explore innovative forms of artivism, having as a starting point the practices of Campos. Through her artistic creations, we seek to understand: (a) how she uses her body and performance to build narratives of resistance; (b) the way in which these elements interact with migration experiences and the Latin American sociocultural context. The choice of Campos came after we had contact with another migrant artist who’s her friend, namely Jazmín Giordano, who lives in the city of Porto, in Portugal. She told us about Campos’ work, and we considered that it would be important for us to broaden our research, to obtain a more comprehensive perspective on feminist artivism and international migratory movements, especially those from the Global South.

Qualitative and/or visual content analysis, from our analytical lens, can be seen as a technique that combines specificities of other methodologies, such as ethnography or grounded theory framework (Timmermans and Tavory, 2007). Some authors say that the content analysis technique was first associated with quantitative methods, as it was intended to objectify and systematize information. Currently, in its broad definition, content analysis refers to a process of categorizing content with a similar and/or complementary meaning (Cho and Lee, 2014, p. 3).

The relation of this technique with quantitative methods was, however, widely criticized under the aegis of the argument that discourses lost their meaning. It was argued that texts and discourses should be interpreted holistically, something that ended up being internalized, with the use of this technique in relation to qualitative methodologies.

The use of this technique, when analyzing the interview and Campos’ performances, allowed us to interpret a series of latent contents and their meanings, as well as opened a field of possibilities that led us to question the relevance of other themes, such as the body in the digital universe. It should be noted that the content analysis technique allows flexibility in the use of an inductive or deductive perspective (Elo and Kyngäs, 2008).

With this in mind, we adopted an inductive model, given that the categories of analysis of the artistic and visual materials and interview were created from the data themselves. We talk about categories such as: artistic processes and experiences; experiential contexts of migration in relation to the arts; dynamics and dimensions of artivism; relationship between territoriality and art; the body as performance and as territory. These categories were later worked on in programs such as Excel (artistic work) and NVivo (interview), to carry out a diachronic and synchronic content analysis.

It remains for us to establish some considerations about the use of a visual sociology, which was used in synchrony with the semi-structured interview: a visual content analysis. Zuev and Krase (2017) and Pauwels (2010) tell us that one of the basic ideas of visual sociology is that it is possible to obtain a scientific view of society, or of a certain social phenomenon, through the observation and analysis of visual manifestations. In the case of the photo-performance *Vaciarce* (2019) and the video-performance *Árido* (2020), we get a glimpse of contemporary creative processes linked to the digital field, the relationship of the female body with the territory, and also a perspective on an emerging typology of artivism. Adding to this perspective, it can be mentioned that visual sociology has already looked at different themes, such as social class, gender, nationalism or multiculturalism, hence, for Zuev and Krase (2017, p. 3).

“The variety of social issues that have been studied within visual sociology demonstrates the need to normalize visual analysis as an integral part of modern sociological research. Such normalization also means that to prove most efficient, methods of visual sociology should be utilized in conjunction with other methods of social enquiry.”

## 3 Soft artivism. Contemporary ways of resisting and of creating

Understanding contemporary artivist practices implies that we are talking about lived experiences and that without them there is no artivism, since this practice starts from a creative intimacy of interactions. At the same time, in Campos’ artistic practices, we find a strong spatial component, both from a physical and symbolic perspective. Her artistic practice begins when Campos decides to pursue an artistic career, since art only appears as a professional path during adulthood.

In the case of women artists, what we have sought to demonstrate with other research done (Sousa, 2024), is that artivism is often used by women artists not as a form of criticism or rupture with a political power, but rather as a symbolic form of claiming a social and emotional space that is sometimes taken away from them, due to structural problems, such as racism, sexism, xenophobia, anti-multiculturalism, etc. We advocate that we are analysing practices and products which materialize a form of soft artivism, and Campos is an example.

To define the concept of soft artivism we depart from Warner (2021) conceptions. The author refers in his work to the concept of soft activism (Warner, 2021, p. 46), based on Mitchell (2018) contributions on soft speech and Mayeri (2008) concept of soft science. The concept of soft artivism enunciated by us refers to an artistic practice with an activist nature, but which is guided by informality, experimentation and accessibility: characteristics that mark Campos’ artistic works and that are explored later in relation to both the performances being analyzed.

The main objective of soft artivism is to cross the traditional boundaries of an artivist practice, namely the fact that it is related to urban art and spaces, that it is ephemeral and that it has a strong political message (Aladro-Vico et al., 2018; Guerra, 2022). This concept portrays a type of practice that is porous to disciplinary boundaries, but also to sensory materialities (Warner, 2021). While we must allude to the fact that the concept of soft artivism has never been used/applied in the social sciences, namely in sociology, it is also an innovative conceptual approach to portray contemporary artivist practices.

Raposo (2022) and Ribas (2014) enunciate the creation of a political vocabulary in which the artivist practice is situated. Political vocabulary refers to the opening of a space where politics can be discussed, based on individual actions. Soft artivism emerges as an aesthetic mechanism that is used to access micro-personal processes of transformation of the intimate sensitive (Rancière, 2005). There is a departure from the typical conceptions of resistance related to the notion of subalternized bodies (Raposo, 2022, p. 7–8). In soft artivism, we argue there’s an emotional urgency and a singular questioning (Raposo, 2022) which requires the creation of an aestheticization as we see in Campos productions, and which abandons the notion of counterpower, struggle and criticism in the face of a system. Her artistic work moves away from the issue of a public manifestation (Giovani, 2015) and approaches intimate and personal spaces.

Artists such as Campos would not - from the outset - be seen as having an artivist practice, especially because her works and artistic creations do not report emerging political situations, economic and housing crises, or even socially localized problems (femicide, for example). However, when we listened to the explanation of the experiences that underlie her artistic creations, we found *other* political spaces, albeit they are non-politicized, namely the issue of mental health, or even the daily experience of being a woman and her connection with the territories, in which a migratory path is evident. From her interview we state that:

I started studying art in 2012, I wasn’t young when I started studying. Then I had to do my thesis and did not want to paint because I started wondering about other stuff, of bodies, what a body means, a lot of questions regarding that, and it was just like I started doing things with my body. I started doing actions privately from that moment until now (Tuty Moreno Campos, 39 years old, Mexico, interview).

While studying at the university, one of her sisters had a health problem and had to be hospitalized, being placed in a minimal state of consciousness. It is from this experience that Campos begins to look for other ways to express herself artistically, since until then she had only painted. Campos begins to question herself about the meanings of the body and what it represents, and it’s at this moment that Campos begins to use her body to create performances and, through them, tries to understand how individuals are affected by the physical environments that surround them. Campos tells us about the need to listen to her body and, at that moment, her body becomes an instrument that promotes an artistic practice.

Another curious aspect concerns the temporalities and spatialities of Campos soft artivism, which are in line with the dimensions of informality and experimentation, proposed by Warner (2021). Since Campos works mainly with video performance, this means that when she is invited to exhibitions or other events, to present her artistic works, her physical body is not present. As she tells us:

I participated in other events but it’s also kind of strange, because most of the times since I was traveling, I participated with some pieces and with some videos, and I wasn’t there. Maybe I would send something, and it would happen after I left the place or something like that. (Tuty Moreno Campos, 39 years old, Mexico, interview).

Authors such as Nercam et al. (2023, p. 9) state that activist artists intend to cultivate disjunction and produce emotional shocks that aim to break with pre-existing social norms. Behind these practices it exists political, ethical and ideological assumptions of action that, in turn, demarcate physical spaces of action. However, Campos and her artistic creations aim to create informal spaces for reflection and coping strategies, often related to the difficulties she faces in maintaining an artistic career. The artivist practice, in this case, starts from a personal space of change which, in turn, is open to experimentation. This is the main argument we make which leads us to understand her artistic work as a form of soft artivism, i.e., artistic creation as a coping mechanism, marked by informality.

An important point, exposed in the interview by Campos, refers to the fact that to create an artistic piece, more specifically a performance, she only needs her body. She also states that initially when she started performing, she used photo-performance and started from a more intimate place. In line with the proposed concept of soft artivism, Campos performed as a matter of necessity, out of an emotional urgency. Performance was her escape and there was no intention of publishing and being invited to reproduce the performance in other contexts and institutional spaces. It was rather a reaction of Campos body to the environment and to what she was feeling: “My process is trying to address something I’m feeling and trying to give it a time and a space to address that. Maybe something will change or not, but for me it’s like that.” (Tuty Moreno Campos, interview).

Thus, concepts such as performativity, understood here as the repetition of acts (Butler, 2025), allow us to understand Campos’ body not as a fixed datum, but rather as a dynamic and constitutive process of her identity as an artist and as a woman. This theoretical perspective highlights that, especially in the context of women’s practices, the body becomes a space of resistance and transformation, which dialogs with the idea of soft artivism that we propose here. In this sense, Campos’ performative approach is also aligned with Butler (2013) analyses of the fluidity and social construction of gender, insofar as they highlight the ways in which the body, by manifesting itself in a ‘performative’ way, does not reproduce fixed stereotypes, but rather subverts normative expectations.

In this regard, Carnevale et al. (2007) state that an artist who is an activist aims to propose modifications to what is visible, in the sense that she finds and provides new ways of perceiving and expressing social reality. Nercam et al. (2023) articulate the idea that an artivist faces several challenges that go beyond denunciation as their practice is involved in opening themselves – and society – to possibilities of alternative action and experience. The authors add to this premise the notion of dialog, aspirations and utopias, and although we also agree with this definition, we think that it becomes too restricted because it places an excessive weight on practices and products with an artivist background, portraying them as if they were a means of salvation or the only possible form of change.

The soft artivism of artists like Campos deals with changes on a micro scale, or even in an individualized panorama. However, in our opinion, they are still a vehicle for change. Often, what happens is that these practices and products have a bigger impact on a smaller scale, that is, when we talk only about the individual who creates these visual narratives, or those who cross paths with them. In addition, it seems to us that there is a biased view in the statement of Nercam et al. (2023). After all, who ‘consumes’ artistic products with an artivist bent? Who creates them and for what contexts and places are they created? Are all artists activists? How do you choose the activist messages that are transmitted? These are some points to think about.

## 4 *Vaciarse.* Tuty’s body between everything and nothing

After this initial framing of the importance of the body in Campos artistic work, we enter the analysis of the photo-performance *Vaciarce* (2019). This artistic work happened during the pandemic, and through it we see a connection between the artist’s body, her mind and the connection with the physical space.

According to the artist

In the case of Vaciarce it was connected to COVID, because I usually have to-do lists and at that moment I had a stack of sticky notes, and I would write what I had to do, and during COVID, I was thinking a lot more, it was like you have all these thoughts because you are all day at moment. I started writing everything that comes to my mind. It wasn’t like everything that came to my mind, because when I started doing it, suddenly my mind it was like empty. I did not know what to write. I was trying to write them down [her thoughts], but at a certain point I was trying to do the opposite, to have or to create a thought. I was trying to take everything from the inside to the outside and put it like an extra skin maybe. And in nature, because we had been in lockdown, and that was the first time that we traveled (Tuty Moreno Campos, 39 years old, Mexico, interview, our highlights).

*Vaciarce* means ‘to empty’, in a literal translation, but in Campos’ case, it reflects a process of liberation. In the images we see Campos body covered with stickers full of thoughts. At this level, we can establish a parallel with the contributions of Dias and Mendieta (2022), as the author mentions that performance is a necessity because it opens a different possibility of relating to the world. We are facing a way of *making* art that also equates different types of ‘being’ from the individual in the world and relating to their thoughts and the surrounding physical space.

Campos body, in this photo-performance, acquires new contours and meanings because it becomes a metaphor of her mind. In this regard, it is relevant to rescue the contributions of Bordo (1997), when he states that the body is also an agent of culture, since it entails several symbolic dimensions of interrelationship with other individuals, but especially with the surrounding environment. This is linked, albeit subjectively, to what Campos told us, about the fact that she approached the issue of stickers with her thoughts as if they were a second skin. This observation relates to Foucault (1998) contributions, since the author argues that the social and cultural contexts are marked in the physical body. This photo-performance allowed this visualization, as well as it allowed the sociological exploration that we do here.

Dias and Mendieta (2022, p. 8, our translation) makes some notes that seem to make this artistic work of Campos explicit,

When art takes the body as matter, to free it and make transparent the tricks of social powers about it, it is transformed and feelings are resignified. It is possible to feel my body through the body of the other, it is possible to feel in my body what I see being done in the body of the other. The experience promoted shocks, frightens and sensitizes. What is happening to the other’s body becomes visibly palpable, common, universal for those who observe, since those who see also have and feel in their body, their own pains, their skin, nerves, blood.

Returning to the concept of soft artivism, i.e., to those three dimensions initially presented by Warner (2021), namely informality, experimentation, and accessibility, we add the dimension of performativity. When we refer to performativity, we can use the figures presented above as an illustration, where we see the contrast of the bright colors of the stickers with gray sand, or even Campos thoughts, as we can read in the stickers: “knowing how to react,” “being able to show weakness,” “tired of being responsible.” Some of Campos’s thoughts also denote some connections with the gender issue, albeit informally, and this can be analyzed in the light of soft artivism because it focuses on awareness-raising and entails a gentle/intimate approach to the subject. Campos also makes some considerations about the difficulties of being a woman and having an artistic career, and in her case, these are reflected in three levels: age, nationality, and type of artistic practice. The excerpt below reveals that the challenges faced by Campos are configured in multiple dimensions: the instability of opportunities, the barriers imposed by institutional mechanisms of inclusion that can become exclusionary, and the selective valuation based on age. These elements demonstrate the complexity of implementing artistic practices as a form of resistance and political participation but also demonstrate that continuing to create artistically is a form of soft artivism. She mentions the following:

During the pandemic I got to know a guy that works in a place and with video, and he invited me to present a video-performance in a series they had. At the beginning it was going to be online and that it would have more visibility, but all the sudden they changed their agenda, and the space had to be allocated to other people. I had to change everything…you must fit that quota because I was Latin. I also think age is an important factor, there’s always these young artists and mid-career artists, and it’s strange but it’s difficult for people to understand, because maybe I’m not young but my career is. If you are older, they do not care if you started a bit late. (Tuty Moreno Campos, 39 years old, Mexico, interview).

## 5 *Árido.* Tuty’s body in relation to land

In *Árido* [Barren], Campos body becomes in tune with the land and with the environment. We also associate this performance with a manifestation of soft artivism, especially for its experimental character. The performance was made and thought while the artist was living in France. In her words,

This is a land of France that they are trying to reforest, it’s very arid and it has a lot of land. We went to visit it a few times, and this is a hostile environment, it’s really…there’s a lot of dust and you do not have internet; there’s a lot of snakes and stuff that may kill you. You have these kinds of very windy days that you cannot go outside because the wind and the dust is too much, in that case it was kind of a poetic though taken to the place like something is not comfortable, what do you do? You try to avoid it? Or something like is you capable of doing the effort to stay there and to be there, even if you do not get connected to nature? Are you able to stay when something it’s not comfortable? (Tuty Moreno Campos, 39 years old, Mexico, interview).

**Figure 1:**
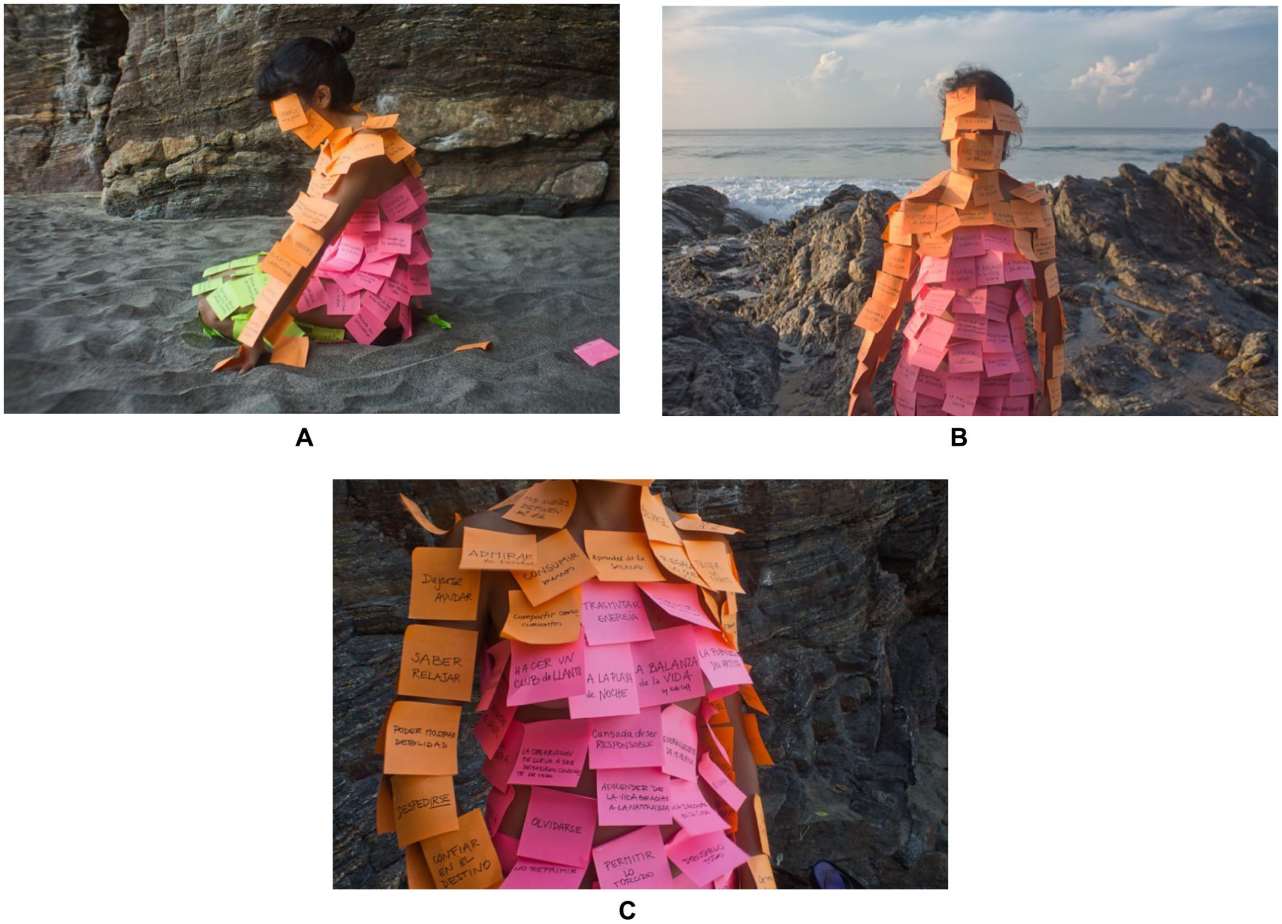
**(A–C)** Records of the photo-performance Vaciarce, by Tuty Moreno Campos, in 2019. Source: Provided by the artist.

As we can read in the excerpt from the interview, we see the physical environment as a manifestation of affective meanings and questions on the part of the artist. Here, in our analysis, we have added one more axis to the dimensions that make up the concept of soft artivism, namely the concept/notion of resistance. In addition, the message that Campos conveys, when she says that there are many natural elements that could kill her, creates a symbolic imaginary that is related to her migration trajectory, and in parallel, to the gender problem (Figure 1).

We decided to choose this specific performance, because it brings with it numerous questions. After talking to the artist, she poses the questions present in the excerpt, that is, the one of questioning what we would do in a situation where our body feels uncomfortable. It is almost inevitable that we do not think about our trajectory and our body in action. What is relevant, in our view, is the fact that the artist approaches her body - at the core of this performance - as an entity, i.e., as a layer that ‘lives’ beyond her consciousness. Let us see photographic registers from the video-performance below.

**Figure 2:**
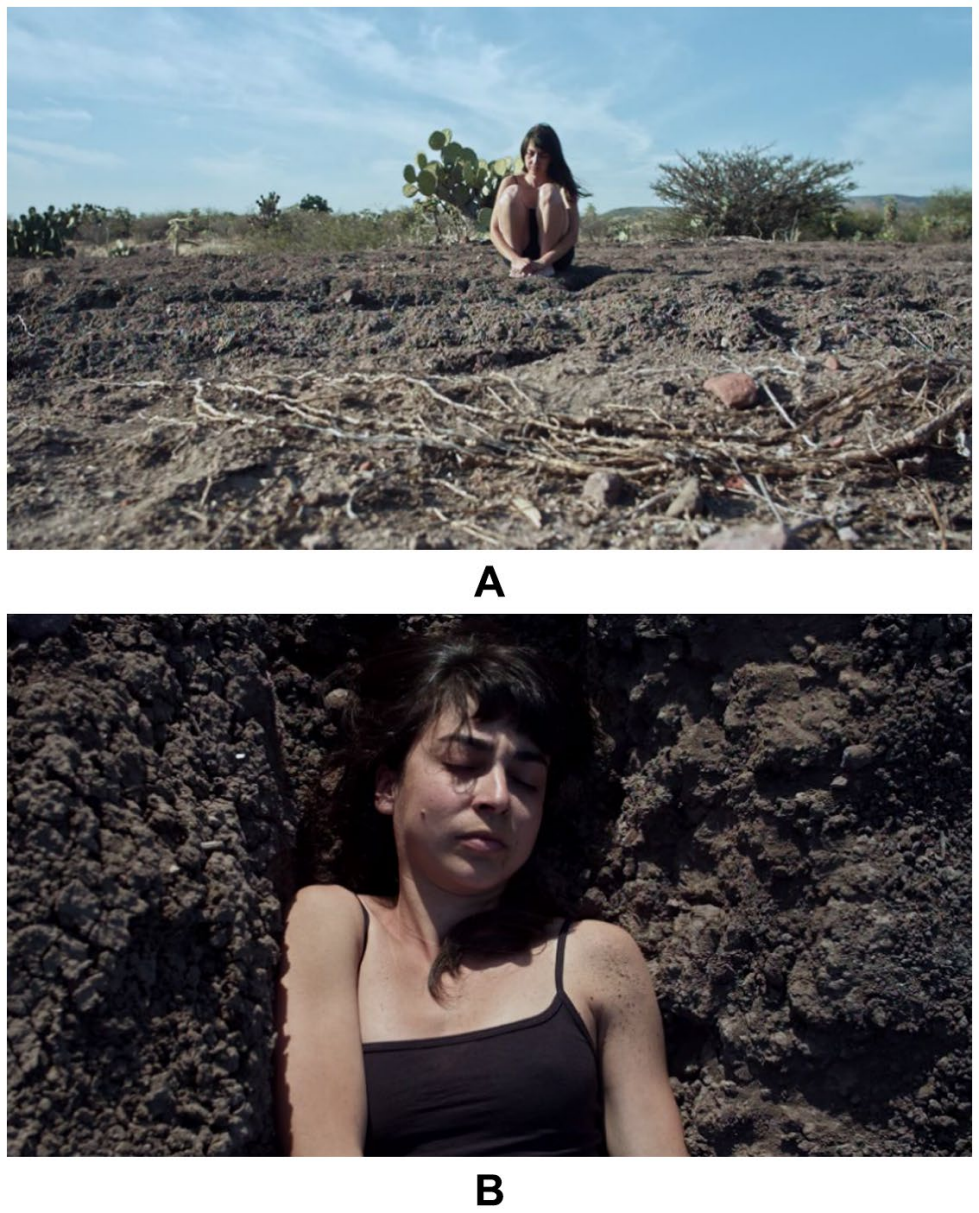
**(A,B)** Records of the video performance Árido, by Tuty Moreno Campos, in 2020. Source: Provided by the artist.

Souza (2019) offers some considerations about the concepts of materiality and embodiment that seem pertinent to us to put this performance by Campos into perspective, mainly because the author mentions that the body is a catalyst for renewed feminist discourses. The images (Figure 2) of the video-performance *Árido* (2020), portray the claim of the situationality and positionality of the female body, since it goes beyond the dualism mind *versus* body. The performance is almost like a ritual. In Figure 2, Campos sits on the ground, almost in a fetal position, conveying the discomfort of her body’s interaction with the land, which, as we can see, is arid, dry and full of lifeless branches. In Figure 2, she lies down in a kind of ditch, resembling a burial.

In *Árido* we see an aesthetic-political position where the body is seen as a vehicle for resistance. There is an emphasis on the female body that allows us to rethink the biopolitical strategies of domestication of the bodies and the territories (Stubs et al., 2018). The artist’s video-performance can also be read as a critique of the capitalist powers, and this is because the physical context where the performance was held was being reforested. By lying down in the ditch, albeit symbolically and in a gentle approach, the artist questions the freedom of the body, seeking to cause a “subversion of identity, a reappropriation of the body as a territory of existence and the inclusion of perspectives of analysis that proceed through a politics of difference” (Stubs et al., 2018, p. 2).

Soft artivism is evident in this performance and it has connections with the concept of feminist aesthetics, coined by Bovenschen (1985). The notion of feminist aesthetics refers to a typology of artistic productions that has an interventional character as an aesthetic strategy of subversion and resistance. This is not so much related to aesthetics or feminist movements, but rather to manners of production and creation that have an interventionist character, but on a scale situated in a micro dimension of social reality. Thus, feminist aesthetics - or aesthetic-political artivism - have a more comprehensive character of action, with a strong political component/message of contestation. In fact, the concept of soft artivism intends to create news patterns of expression of corporeality that go beyond traditional artivism in which the physical bodies are also used.

## 6 The aftermath of artivism

The analysis advocated has an exploratory character, in the sense that it aims to address – in an initial and superficial logic – the concept of soft artivism. Firstly, it is important to highlight that we start from the contributions of Warner (2021), regarding the concept of soft activism, which is closely linked to the issues of interior design and urban space design. The concept of soft activism is much more an explanation of the relationships between individuals and physical spaces, in a logic of interaction and affective meaning. However, Warner (2021) indicated a set of characterizing axes that, from our perspective, can be adapted to describe artistic practices such as Campos’.

*Vaciarce* (2019) and *Árido* (2020) are artistic practices that have an activist nature and that are characterized by informality, experimentation, and accessibility. The informality of both performances comes from the fact that they are created and presented while the artist is in motion, that is, there is no link or need to have them represented in institutional spaces. Informality comes from the mobility that guides the artist’s experience, that is, from her migratory paths. On the other hand, the issue of experimentation is articulated with the use of her body as a recipient of experiences related to physical spaces, but also with the exploration of mental and affective states (gentle protest). Accessibility happens by mobilizing digital media as a demonstration vehicle.

To the three dimensions presented above, we have added one more, namely those of performativity. Performativity emerges here as a typology of artistic creation. In our opinion, the concept of soft artivism needs to have a component of visual and physical representation of the creative body itself. Soft artivism implies actions.

We decided to explore theoretically and empirically the concept of soft artivism given the specificity of the artist’s artistic practice. In fact, when carrying out the state of the art/literature review, we found numerous articles that cross the themes of feminism, performance and artivism, however, none of them could be applied to a reading of Campos’ artistic works. Direct political messages referring to emerging social problems are not present, there is no component of urban spatiality or even public demonstrations. Campos creations do not have an ephemeral character nor are they averse to the institutionalization of aesthetic-political artistic creations.

Although *Vaciarce* and *Árido* do not fit the typical characteristics and definitions of what is considered an artivist practice, from our perspective, they still have an artivist purpose. Political messages/readings are present, they only portray a micro, individual and affective scale. We are faced with a set of artistic works that go beyond conventional readings on performativity, body and artivism and that, therefore, need to be explored in other theoretical-conceptual dimensions.

## Data Availability

The original contributions presented in the study are included in the article/supplementary material, further inquiries can be directed to the corresponding author.

## Footnotes

^1^The author(s) disclosed receipt of the following financial support for the research, authorship, and/or publication of this article: this article received funding from the Foundation for Science and Technology (FCT), corresponding to an individual PhD scholarship entitled ‘All Worlds within Porto. Migrant women, arts and artivism in contemporary Portugal’. (https://doi.org/10.54499/2021.06637.BD). The publication was supported by the FCT – Foundation for Science and Technology, within the scope of UIDP/00727/2020.

^2^Her work can be consulted at: https://tutymorenocampos.com/
